# Factors Associated With Progression, Resolution and Mortality of Patients With Overt Hepatic Encephalopathy

**DOI:** 10.1016/j.jceh.2025.103410

**Published:** 2025-11-07

**Authors:** Maria P. Ballester, Anindro Bhattacharya, Ferran Aguilar, François Fenaille, Cristina Sánchez-Garrido, Richard Moreau, Vicente Arroyo, Victor Vargas, Wim Laleman, Joan Clària, Jonel Trebicka, Juan A. Carbonel-Asins, Christopher F. Rose, Rajiv Jalan, Predict Study Group and AMMON Consortium

**Affiliations:** ∗ Digestive Disease Department, Hospital Clínico Universitario de Valencia, Spain; † INCLIVA Biomedical Research Institute, Valencia, Spain; ‡ University of Pennsylvania, Department of Bioengineering, Philadelphia, United States; § European Foundation for the Study of Chronic Liver Failure (EF CLIF), Barcelona, Spain; ¦ Université Paris-Saclay, CEA, INRAE, Département Médicaments et Technologies pour la Santé, MetaboHUB, Gif-sur-Yvette, France; ¶ INSERM, Université Paris Cité, Centre de Recherche sur l’Inflammation (CRI), Paris, France; # Assistance Publique-Hôpitaux de Paris (AP-HP), Hôpital Beaujon, Service d’Hépatologie, Clichy, France; ∗∗ Facultad de Medicina, Unidad docente Vall d’Hebron, Universidad Autónoma de Barcelona, Spain; †† Department of Gastroenterology & Hepatology, Division of Liver & Biliopancreatic disorders and Liver Transplantation, University Hospitals Leuven, KU LEUVEN, Leuven, Belgium; ‡‡ Hospital Clínic de Barcelona, Institut d’Investigacions Biomèdiques August Pi i Sunyer, Universitat de Barcelona, Centro de Investigacion Biomedica en Red de Enfermedades Hepaticas y Digestivas, Spain; §§ Department of Internal Medicine B University Clinic Münster, Germany; ¦¦ Hepato-Neuro Laboratory, CRCHUM, Université de Montréal, Montreal, Canada; ¶¶ Institute for Liver and Disease Health, University College London, London, United Kingdom

**Keywords:** overt hepatic encephalopathy, acute-on-chronic liver failure, cirrhosis, prognosis, natural history

## Abstract

**Background:**

Overt hepatic encephalopathy (OHE) is a reversible complication of cirrhosis that often results in hospitalization. Factors associated with progression, resolution and mortality are not known, particularly with confounders such as acute-on-chronic liver failure (ACLF). The aim of the study was to evaluate factors associated with progression, resolution, and mortality of patients with OHE.

**Methods:**

Data for this study were derived from PREDICT, a prospective cohort study of patients with cirrhosis hospitalized for an acute decompensation or ACLF. Progression to OHE or worsening in severity and resolution from OHE were evaluated at 1 week. Cox regression, interaction analyses, and Kaplan–Meier curves were performed.

**Results:**

One thousand two hundred seventy-three patients were included [68% males; 59 (51–67) years; 56% alcohol], 16% admitted with OHE and 16% with ACLF. Older age, metabolic dysfunction-associated steatotic liver disease, previous treatment with lactulose, ACLF, white blood cell counts or albumin levels at admission were associated with OHE (*P* < 0.05). OHE progressed in 3% patients, which was associated with older age, previous treatment with lactulose and bacterial infections (*P* < 0.05), with a significantly shorter time-to-death (*P* < 0.001). Patients who resolved OHE (79%) presented a similar prognosis than those without OHE (*P* = 0.208). *Post hoc* analysis of the age-adjusted interaction between OHE and ACLF to predict mortality showed higher differences across ACLF grades compared with OHE.

**Conclusion:**

Presence of ACLF and progression of OHE are associated with high short-term mortality rates, while resolution of OHE is associated with significantly better prognosis. Understanding the natural history of OHE will have profound implications on the development of novel approaches.

## Graphical abstract

**Figure:**
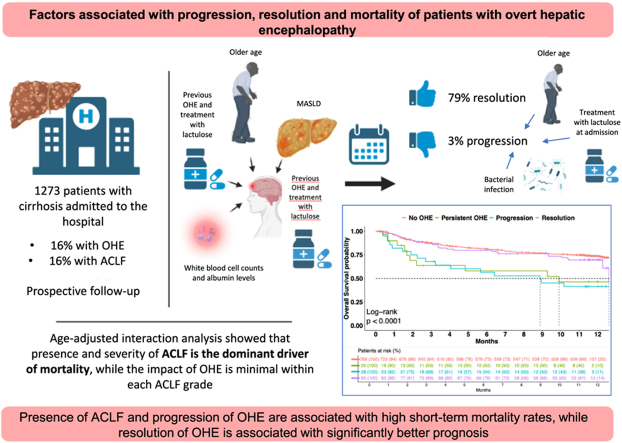

Overt hepatic encephalopathy (OHE) is a potentially reversible complication of cirrhosis, which occurs in about 30–45% of patients with cirrhosis and up to 50% of patients with transjugular intrahepatic portosystemic shunt, and in most of the cases with several relapses.^1^, ^2^, ^3^, ^4^ These patients often need to be cared on intensive care units and have a high risk of short-term mortality.^5^^,^^6^ In addition, the burden of OHE is increasing, as indicated by increases in hospital admissions and higher costs per stay.^7^^,^^8^

There are several well-recognized factors associated with the occurrence of an acute episode of OHE. The most prevalent are infection, gastrointestinal bleeding (GIB), electrolyte disorders/dehydration or constipation.^1^^,^^2^^,^^9^ Identifying and treating these precipitating factors is the primary intervention in patients with OHE as this has been shown to improve OHE in 90% of cases.^10^ This is essential because the absence of improvement or progression of HE has been associated with an increased risk of death.^6^ Nonetheless, factors influencing progression and resolution of OHE as well as mortality remain poorly understood, and are barely modified, particularly with confounders such as the presence or absence of acute-on-chronic liver failure (ACLF), use of specific therapies and lack of accurate follow-up data. Therefore, the aims of the study were to evaluate the natural history of an acute episode of OHE and to determine factors associated with progression, resolution, and mortality.

## MATERIALS AND METHODS

### Study Design and Patient Selection

The data for this study were from the PREDICT study, which was a prospective, observational cohort study of patients with cirrhosis hospitalized for an acute decompensation (AD) or ACLF in 48 hospitals in Europe.^11^^,^^12^ The diagnosis of cirrhosis was based on previous liver biopsy or a composite of clinical signs and findings provided by laboratory test results, endoscopy, and ultrasonography. Diagnostic criteria for AD were based on the development of ascites, OHE, GIB, infection, or any combination of these (definitions are included in the supplementary material). ACLF diagnosis was based on the European definition.^13^^,^^14^ OHE diagnosis was clinically based according to guidelines,^15^ and its evaluation at the baseline and at week 1 was a prespecified outcome at the conception of PREDICT and was prospectively performed at all the study sites. Progression of HE was defined as development of OHE (grade 2 or higher according to the West Haven criteria) or transition from grade 2 to grade 3 or 4 at week 1. OHE that did not improve nor progress at week 1 was defined as persistent OHE. Resolution of OHE was defined as the absence of OHE at week 1 in patients with OHE at admission. Clinical parameters, as well as biochemical and inflammatory markers were evaluated at the baseline and week 1 for the analysis of factors associated with HE evolution. Measurement of inflammatory markers is described in the supplementary material. As per the original protocol, only patients without ACLF at admission were evaluated at week 1 for progression or resolution of HE. Patients who were not evaluated at week 1 or those who were lost to follow-up before 28 days were not included in the analysis of factors associated with OHE progression, resolution, or 28-day mortality, respectively. Patients were prospectively followed until transplantation or death up to 1 year after enrollment. A flowchart of the study population is shown in Supplementary Figure 1.

### Statistical Analysis

Normally distributed data were reported as mean and standard deviation and were compared using the Student’s t test or analysis of variance while non-normally distributed data were reported as the median and interquartile range (IQR) and were compared using the Wilcoxon signed-rank test or the Kruskal–Wallis test. Categorical data were reported as number and percentage (%) and comparisons were analysed by Chi-squared (*χ*^2^) test or Fisher’s exact test when expected cell count was less than 5.

Univariable and multivariable logistic regression analyses were performed to evaluate factors associated with progression or resolution of HE. All survival models were performed using Cox regression censoring for patients at either 28 days or end of follow-up accordingly. Multivariable model selection was fitted by using a stepwise backward method based on Akaike information criteria using all variables included in a univariable analysis. Severity scores such as Child–Pugh or model for end-stage liver disease (MELD) scores, original variables that are part of the ACLF or electrolyte disorder definitions (included in supplementary material) as well as history of HE was not included to avoid collinearity. Survival was evaluated with Kaplan–Meier curves and the log-rank test, Benjamini–Hochberg *P* value adjustment was carried out when multiple survival curves were compared.

Cox regression including age, ACLF, and HE was performed to evaluate the interaction effect between HE and ACLF on the risk of death. *Post hoc* analysis of the interaction was adjusted for multiple comparisons using the Bonferroni method.

All analyses were performed with R (version 4.0.2, R Core, 2021) with the cut-off for statistical significance set at 0.05.

## RESULTS

### Study Population

A total of 1273 patients were included in the study; 68% were males and the median age was 59 (IQR: 51–67) years. Patient characteristics are described in Table 1. Most of the patients (97%) were Caucasian. Alcohol was the most prevalent etiology of cirrhosis (56%). A total of 205 patients (16%) presented with OHE. The most frequent precipitating illness at admission were bacterial infection (33%) followed by electrolyte disorders (32%). A total of 202 (16%) patients were hospitalized with ACLF. Liver (75%) and kidney (63%) failures were the most frequent organ failures, with brain failure present in 63 patients (31%). A total of 174 (14%) patients underwent liver transplantation and 409 (32%) died at the end of the study period.

**Table 1:** Characteristics of the Total Cohort and Patients With or Without OHE at Admission.

Parameter	Total (n = 1273)	No-OHE (n = 1068)	OHE (n = 205)	*P*-value
Male sex, n (%)	867 (68)	725 (68)	142 (69)	0.697
Age, median (IQR)	59 (51–67)	58 (51–66)	60 (52–70)	0.006
Race, n (%)				0.063
Caucasian	1232 (97)	1035 (97)	197 (96)	
Black	5 (0.4)	2 (0.2)	3 (2)	
Asian	16 (1.3)	14 (1)	2 (1)	
Other	20 (1.6)	17 (2)	3 (2)	
Etiology of cirrhosis, n (%)				
Alcohol	716 (56)	609 (57)	107 (53)	0.557
HCV	81 (6)	66 (6)	15 (7)	0.488
Alcohol + HCV	66 (5)	50 (5)	16 (8)	0.069
MASLD	96 (8)	79 (7)	17 (8)	0.569
Other	314 (25)	264 (25)	50 (24)	
Previous history (3-month), n (%)				
Ascites	600 (47)	498 (47)	102 (50)	0.141
HE	236 (19)	135 (13)	101 (49)	<0.001
GIB	132 (10)	113 (11)	49 (24)	0.576
Hospitalization for infection	183 (14)	114 (11)	39 (19)	0.041
SBP	60 (5)	46 (4)	14 (7)	0.102
ACLF	63 (5)	50 (5)	13 (6)	0.315
Treatments, n (%)				
Lactulose	707 (56)	530 (50)	177 (86)	<0.001
Rifaximin	300 (24)	218 (20)	82 (40)	<0.001
HE grades, n (%)				
0	834 (66)	834 (78)	0 (0)	NA
1	234 (18)	234 (22)	0 (0)	
2	142 (11)	0 (0)	142 (69)	
3	48 (4)	0 (0)	48 (23)	
4	15 (1)	0 (0)	15 (7)	
Precipitating illness at admission, n (%)				
Electrolyte disorder	406 (32)	342 (32)	64 (31)	0.855
GIB	186 (15)	161 (15)	25 (12)	0.285
Bacterial infection	421 (33)	339 (32)	82 (40)	0.021
Alcohol use disorder	201 (16)	167 (16)	34 (17)	0.737
Other	275 (21)	145 (14)	130 (63)	<0.001
ACLF at admission, n (%)	202 (16)	137 (12)	75 (37)	<0.001
ACLF grade, n (%)				
0	1067 (84)	937 (88)	130 (63)	<0.001
1	119 (9)	92 (9)	27 (13)	
2	58 (5)	30 (3)	28 (14)	
3	25 (2)	5 (1)	20 (10)	
Organ failure, n (%)				
Liver	152 (12)	124 (12)	28 (14)	0.410
Kidney	128 (10)	107 (10)	21 (10)	0.922
Brain	63 (5)	0 (0)	63 (31)	<0.001
Coagulation	80 (6)	52 (5)	28 (14)	<0.001
Circulatory	32 (3)	8 (1)	24 (12)	<0.001
Respiratory	13 (1)	4 (0.4)	9 (4)	<0.001
Child–Pugh class, n (%)				
A	86 (7)	83 (8)	3 (2)	<0.001
B	546 (43)	478 (45)	68 (33)	
C	526 (41)	403 (38)	123 (60)	
Unknown	115 (9)	104 (10)	11 (5)	
Child–Pugh, median (IQR)	9 (8–11)	9 (8–10)	10 (9–12)	<0.001
MELD, median (IQR)	17 (13–22)	16 (12–21)	18 (15–25)	<0.001
MELD-Na, median (IQR)	20 (15–25)	20 (15–24)	22 (17–27)	<0.001
CLIF-AD score, median (IQR)	52 (47–57) (n = 1071)	52 (47–57)	54 (50–58)	0.007
CLIF-ACLF score, median (IQR)	48 (44–54) (n = 202)	47 (42–51)	54 (48–61)	<0.001
Biochemical parameters, median (IQR)				
WCC (x10ˆ9/L)	6.7 (4.5–9.5)	6.7 (4.4–9.4)	6.9 (5.0–10.5)	0.024
Platelets (x10ˆ9/L)	99 (63–148)	100 (64–152)	92 (59–127)	0.021
INR	1.5 (1.3–1.8)	1.5 (1.3–1.7)	1.6 (1.4–2.0)	<0.001
Prothrombin time (seg)	17 (14–23)	17 (14–22)	18 (15–27)	<0.001
Albumin (g/dL)	2.8 (2.5–3.3)	2.9 (2.5–3.3)	2.8 (2.5–3.1)	0.051
AST (U/L)	56 (35–93)	56 (34–92)	56 (39–94)	0.272
ALT (U/L)	29 (19–46)	29 (19–46)	33 (21–50)	0.016
Bilirubin (mg/dL)	2.9 (1.5–6.7)	2.8 (1.4–6.5)	3.3 (1.9–7.7)	0.002
Creatinine (mg/dL)	0.9 (0.7–1.3)	0.9 (0.7–1.3)	1.0 (0.7–1.5)	0.043
Sodium (mEq/L)	136 (132–138)	136 (132–138)	135 (131–139)	0.607
Potassium (mEq/L)	4 (3.6–4.4)	4 (3.6–4.4)	4 (3.6–4.6)	0.486
C reactive protein (mg/L)	18 (8–40)	18 (8–40)	18 (7–40)	0.575
Inflammatory markers (pg/mL), median (IQR)				
Eotaxin	80 (57–112)	80 (56–111)	81 (58–120)	0.194
G-CSF	17 (6–45)	16 (6–44)	22 (7–49)	0.350
GM-CSF	10 (2–32)	10 (2–30)	9 (2–43)	0.935
IFN-alpha2	15 (4–32)	15 (5–32)	16 (3–30)	0.380
INF-gamma	25 (9–71)	25 (9–72)	24 (8–64)	0.544
IL-1alpha	2.7 (0.9–6.5)	2.8 (0.9–6.5)	2.4 (0.6–6.6)	0.423
IL-1beta	4.7 (2.0–11.5)	4.7 (2.1–11.5)	4.7 (2.0–12.1)	0.918
IL-1RA	3.8 (2.0–8.0)	3.7 (1.9–7.7)	4.3 (2.3–10.7)	0.045
IL-4	1.6 (0.4–4.3)	1.6 (0.4–4.5)	1.4 (0.3–3.9)	0.341
IL-6	11.1 (5.4–26.2)	10.8 (5.3–25.1)	13.6 (6.1–35.0)	0.018
IL-7	1.2 (0.4–3.6)	1.3 (0.4–3.9)	0.8 (0.3–2.8)	0.043
IL-8	4.0 (1.6–10.3)	3.9 (1.6–10.3)	4.4 (1.5–9.8)	0.991
IL-10	6.7 (2.5–17.0)	6.5 (2.3–16.5)	9.4 (2.8–28.7)	0.021
IL-17A	3.0 (1.1–7.2)	3.0 (1.1–7.2)	2.7 (0.9–8.3)	0.866
IP-10	197 (116–350)	194 (116–338)	214 (113–403)	0.327
MCP-1	167 (118–236)	167 (120–234)	167 (110–248)	0.690
MIP-1alpha	13.0 (5.7–23.5)	13.0 (5.9–23.5)	12.4 (5.2–22.5)	0.563
MIP-1beta	15.6 (11.5–21.1)	15.4 (11.4–20.7)	17.0 (11.9–24.5)	0.062
TNF-alpha	24.0 (14.7–42.4)	23.5 (14.7–41.3)	26.9 (13.1–44.7)	0.372
VEGF-A	5.2 (1.7–12.8)	5.2 (1.7–12.8)	5.1 (1.8–12.7)	0.927

*P* value compares patients with and without OHE at admission.

Abbreviations: ACLF, acute-on-chronic liver failure; AD, acute decompensation; ALT, alanine aminotransferase; ASP, aspartate aminotransferase; BMI, body mass index; G-CSF, granulocyte colony-stimulating factor; GIB, gastrointestinal bleeding; GM-CSF, granulocyte-macrophage colony-stimulating factor; HCV, hepatitis C virus; HE, hepatic encephalopathy; IFN, interferon; IL, interleukin; INR, international normalized ratio; IQR, interquartile range; MASLD, metabolic dysfunction-associated steatotic liver disease; MCP, monocyte chemoattractant protein; MIP, macrophage inflammatory protein; OHE, overt hepatic encephalopathy; TNF, tumor necrosis factor; SBP, spontaneous bacterial peritonitis; VEGF, vascular endothelial growth factor; WCC, white cell count.

### Presence of OHE at Admission

Patients with OHE were significantly older (60 vs 58 years old, *P* = 0.006), had history of previous HE (49% vs 13%, *P* < 0.001) and previous hospitalization for bacterial infection (19% vs 11%, *P* = 0.041) compared to those without OHE. Accordingly, patients with OHE were more often receiving lactulose (86% vs 50%, *P* < 0.001) or rifaximin (40% vs 20%, *P* < 0.001) at admission. Bacterial infection was the precipitating illness for admission in 40% and 32% of patients with and without OHE, respectively (*P* = 0.021), while no significant differences were seen in the proportion of patients with electrolyte disorders, alcohol use disorder, or GIB (*P* > 0.05). ACLF was more frequent (37% vs 12%, *P* < 0.001), and disease severity was more advanced in patients with OHE with higher Child–Pugh (*P* < 0.001), MELD (*P* < 0.001), CLIF-AD (*P* = 0.007) and CLIF-ACLF (*P* < 0.001) scores. Patients with OHE presented higher white cell count and creatinine and lower platelets (*P* < 0.05). Among systemic markers, patients with OHE presented higher levels of IL-1RA, IL-6, and IL-10 and lower levels of IL-7 (*P* < 0.05) (Table 1).

In a multivariable logistic regression analysis, the selected model of factors associated with OHE at admission included older age, metabolic dysfunction-associated steatotic liver disease (MASLD) etiology, treatment with lactulose, ACLF, lower albumin levels, and higher white blood cell count at baseline (Table 2). Significant factors associated with OHE at admission remained the same when electrolyte disorder was separated in either hyponatremia or hypokalaemia (Supplementary Table 1), but a higher risk of OHE was observed when multiple concomitant precipitating factors were identified at admission compared with only one (Supplementary Table 2). A subgroup analysis of factors associated with OHE in patients with and without prior HE is shown in Supplementary Table 3.

**Table 2:** Univariable and Multivariable Logistic Regression Analysis of Factors Associated With OHE at Admission and With Progression of OHE at Week 1.

Parameter	Univariable		Multivariable	
	OR (95%CI)	*P* value	OR (95%CI)	*P* value
**Presence of OHE at admission (n=1273)**				
Female sex	0.62 (0.35–1.08)	0.101		
Age	1.03 (1.01–1.05)	0.016	1.04 (1.01–1.06)	0.009
Etiology of cirrhosis, n (%)				
Alcohol				
HCV	0.86 (0.52–1.45)	0.559		
MASLD	0.77 (0.26–1.83)	0.587	2.43 (1.05–5.38)	
Other	2.38 (1.10–4.82)	0.020		0.032
Lactulose	4.59 (2.57–8.76)	<0.001	4.82 (2.61–9.47)	<0.001
Rifaximin	2.29 (1.33–3.88)	0.002		
Electrolyte disorder	0.72 (0.41–1.21)	0.225		
GIB	0.72 (0.31–1.48)	0.399		
Bacterial infection	1.69 (1.03–2.78)	0.037		
Alcohol use disorder	0.76 (0.35–1.48)	0.443		
ACLF at admission	3.59 (2.03–6.23)	<0.001	2.75 (1.43–5.21)	0.002
WCC	1.08 (1.03–1.12)	0.001	1.09 (1.03–1.15)	0.001
Platelets	1.00 (0.99–1.00)	0.128		
Albumin	0.60 (0.39–0.91)	0.017	0.55 (0.34–0.87)	0.011
IL-6	1.00 (1.00–1.00)	0.070		
IL-8	1.01 (1.00–1.01)	0.011		
IL-10	1.00 (1.00–1.00)	0.021		
MCP-1	1.00 (1.00–1.00)	0.001		
**Progression of OHE at week 1 (n=902)**				
Female sex	1.57 (0.50–4.80)	0.429		
Age	1.04 (0.99–1.10)	0.103	1.05 (0.99–1.12)	0.109
Etiology of cirrhosis, n (%)				
Alcohol				
HCV	0.91 (0.30–3.05)	0.865		
MASLD	0.87 (0.05–4.64)	0.897		
Other	0.85 (0.05–4.49)	0.873		
Lactulose	3.30 (0.99–14.88)	0.073	3.97 (1.13–19.08)	0.048
Rifaximin	0.73 (0.11–2.78)	0.683		
Electrolyte disorder	1.80 (0.57–5.51)	0.302		
GIB	0.54 (0.03–2.84)	0.560		
Bacterial infection	2.39 (0.78–7.56)	0.125	2.51 (0.76–8.55)	0.128
Alcohol use disorder	1.62 (0.36–5.47)	0.475		
WCC	0.94 (0.79–1.07)	0.403		
Platelets	1.00 (0.99–1.00)	0.299		
INR	1.06 (0.26–1.86)	0.893		
Bilirubin	1.05 (0.94–1.13)	0.332		
Albumin	1.54 (0.62–3.59)	0.335		
Creatinine	0.82 (0.15–3.53)	0.797		
IL-6	1.00 (1.00–1.00)	0.099	1.00 (1.00–1.01)	0.015
IL-8	1.01 (0.99–1.02)	0.326	1.02 (1.00–1.03)	0.022
IL-10	1.00 (0.98–1.00)	0.867		
MCP-1	1.00 (0.99–1.00)	0.690	0.99 (0.99–1.00)	0.040

Severity scores and original variables that are part of the ACLF definition or electrolyte disorder were not included.

Abbreviations: ACLF, acute-on-chronic liver failure; CI, confidence interval; GIB, gastrointestinal bleeding; IL, interleukin; INR, international normalized ratio; MASLD, metabolic dysfunction-associated steatotic liver disease; MCP, monocyte chemoattractant protein; OHE, overt hepatic encephalopathy; OR, odds ratio; WCC, white cell count .

### Progression of OHE During Hospitalization

Of those patients evaluated at week 1 for the presence of OHE (n = 902), a total of 28 (3%) progressed (25 without OHE at admission that developed OHE and 3 patients with OHE grade 2 at admission that worsened to grade 3 at week 1) and 20 (2%) presented persistent OHE. A higher proportion of treatment with lactulose at admission (75% vs 54%, *P* = 0.026), bacterial infections (54% vs 29%, *P* = 0.005), and alcohol use disorder (32% vs 14%, *P* = 0.011) were seen in OHE patients that progressed. Liver function assessed with the Child–Pugh, MELD, MELD-Na, and CLIF-AD scores was also worse in patients whose OHE progressed (Supplementary Table 4).

In the multivariable analysis, the selected model of factors associated with OHE progression included older age, treatment with lactulose at admission, bacterial infection, and higher levels of IL-6 and IL-8 and lower levels of monocyte chemoattractant protein (MCP)-1 (Table 2). In addition, a time-varying analysis showed that the evolution of a bacterial infection was associated with the risk of OHE development (1% in patients with no infection, 6% in patients with persistent infection, 7% in patients who resolved an infection, and 10% in patients who developed it during follow-up; *P* < 0.001). No relationship was found between OHE progression and the trajectory of other covariates such as GIB or renal dysfunction (*P* > 0.050).

### Resolution of OHE During Hospitalization

Of those with OHE at admission that were evaluated at week 1 (n = 108), a total of 85 patients (79%) resolved. Although there was a higher proportion of patients with HE grades 3 and 4 (30% vs 15%) at admission among those that did not resolve OHE at week 1, differences were not statistically significant (*P* = 0.218). MELD and MELD-Na scores were significantly higher in patients who did not resolve OHE (*P* = 0.039 and *P* = 0.035, respectively) (Supplementary Table 5).

### Factors Associated With Mortality

**Figure 1:**
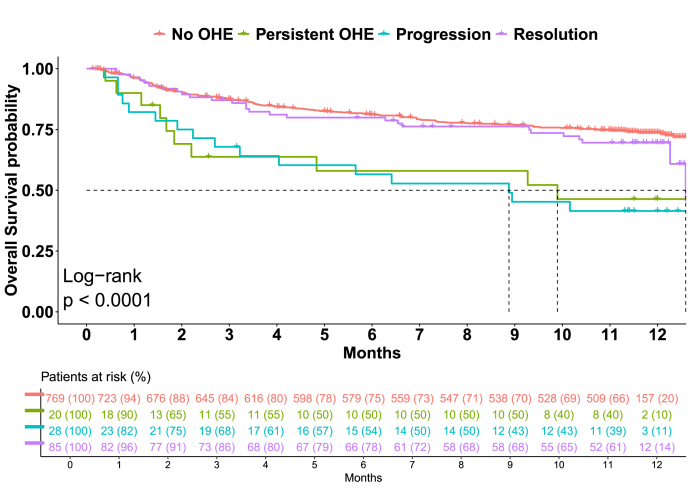
Kaplan–Meier curve of patients without OHE or with OHE progression, persistence or resolution during hospitalization. OHE, overt hepatic encephalopathy.

Overall mortality was higher in patients with than without OHE at admission (48% vs 31% at 12 months) with a significantly shorter time-to-death (log-rank *P* < 0.001), and an increased risk with the severity of HE (log-rank *P* < 0.001). Compared with patients with no-OHE during hospitalization, patients with OHE progression (adjusted log-rank *P* < 0.001), or persistence of OHE (adjusted log-rank *P* = 0.007), presented a higher risk and a shorter time-to-death, while patients who resolve OHE presented a similar prognosis than those without OHE (adjusted log-rank *P*-value 0.208) (Figure 1).

Considering other clinical factors, older age at presentation, history of ascites, HE, or ACLF, treatment with lactulose or rifaximin at the baseline, presence of ACLF at admission, electrolyte disorder or bacterial infection as precipitating illness, more advanced liver disease severity, and higher inflammatory parameters such as white blood cell count, IL-6, or MCP-1, were associated with a higher risk of death during follow-up (Table 3). A multivariable Cox regression analysis in the total population and in the subgroup of patients with or without OHE at admission included age, ACLF, white blood cell counts, and IL-6 levels as the common significant variables associated with the risk of death within 28-days (Table 4).

**Table 3:** Cox Regression Analysis of Factors Associated With Overall Mortality During Follow-up.

Parameter	HR (95%CI)	*P* value
Male sex, n (%)	0.95 (0.77–1.17)	0.600
Age, median (IQR)	1.03 (1.02–1.04)	<0.001
Race, n (%)		
Caucasian	Ref.	Ref.
Black	0.61 (0.09–4.35)	0.623
Asian	0.49 (0.16–1.52)	0.215
Other	0.88 (0.39–1.98)	0.763
Etiology of cirrhosis, n (%)		
Alcohol	1.06 (0.85–1.32)	0.612
HCV	0.66 (0.41–1.04)	0.073
MASLD	1.03 (0.71–1.47)	0.887
Other		
Previous history (3-month), n (%)		
Ascites	1.49 (1.19–1.85)	<0.001
HE	1.63 (1.29–2.06)	<0.001
GIB	0.86 (0.61–1.20)	0.360
Hospitalization for infection	1.38 (1.06–1.79)	0.017
SBP	1.49 (0.99–2.24)	0.055
ACLF	1.99 (1.37–2.88)	<0.001
Treatments, n (%)		
Lactulose	1.31 (1.07–1.59)	0.008
Rifaximin	1.23 (1.07–1.59)	0.008
OHE at admission, n (%)	1.86 (1.48–2.34)	<0.001
HE grade, n (%)		
0	Ref.	Ref.
1	1.43 (1.12–1.84)	0.004
2	1.79 (1.36–2.36)	<0.001
3	2.44 (1.61–3.68)	<0.001
4	3.80 (1.95–7.40)	<0.001
Precipitating illness at admission, n (%)		
Electrolyte disorder	1.45 (1.19–1.77)	<0.001
GIB	0.75 (0.55–1.01)	0.055
Bacterial infection	1.43 (1.17–1.74)	<0.001
Alcohol use disorder	1.13 (0.88–1.46)	0.345
ACLF at admission, n (%)	2.56 (2.05–3.19)	<0.001
ACLF grade, n (%)		
0	Ref.	Ref.
1	1.87 (1.40–2.51)	<0.001
2	3.14 (2.22–4.46)	<0.001
3	8.02 (5.04–12.77)	<0.001
Organ failure, n (%)		
Liver	2.02 (1.56–2.60)	<0.001
Kidney	1.36 (1.02–1.82)	0.039
Brain	2.31 (1.63–3.29)	<0.001
Coagulation	2.48 (1.82–3.38)	<0.001
Circulatory	3.16 (2.02–4.95)	<0.001
Respiratory	5.16 (2.66–10.00)	<0.001
Child–Pugh class, n (%)		
A	Ref.	Ref.
B	1.46 (0.86–2.50)	0.163
C	3.36 (1.99–5.67)	<0.001
Child–Pugh, median (IQR)	1.30 (1.23–1.37)	<0.001
MELD, median (IQR)	1.09 (1.07–1.10)	<0.001
MELD-Na, median (IQR)	1.09 (1.08–1.11)	<0.001
CLIF-AD score, median (IQR)	1.07 (1.05–1.08)	<0.001
CLIF-ACLF score, median (IQR)	1.09 (1.07–1.11)	<0.001
Biochemical parameters, median (IQR)		
WCC (x10ˆ9/L)	1.05 (1.03–1.07)	<0.001
Platelets (x10ˆ9/L)	1.00 (1.00–1.00)	0.206
INR	1.37 (1.28–1.47)	<0.001
Prothrombin time (seg)	1.01 (1.01–1.02)	<0.001
Albumin (g/dL)	0.68 (0.57–0.81)	<0.001
AST (U/L)	1.00 (1.00–1.00)	<0.001
ALT (U/L)	1.00 (1.00–1.00)	<0.001
Bilirubin (mg/dL)	1.05 (1.04–1.06)	<0.001
Creatinine (mg/dL)	1.41 (1.29–1.54)	<0.001
Sodium (mEq/L)	0.96 (0.94–0.98)	<0.001
Potassium (mEq/L)	1.20 (1.05–1.37)	0.007
C reactive protein (mg/L)	1.00 (1.00–1.00)	0.057
Inflammatory markers (pg/mL), median (IQR)		
Eotaxin	1.00 (1.00–1.00)	0.263
G-CSF	1.00 (1.00–1.00)	0.169
GM-CSF	1.00 (1.00–1.00)	0.178
IFN-alpha2	1.00 (1.00–1.00)	0.365
INF-gamma	1.00 (1.00–1.00)	0.523
IL-1alpha	1.00 (1.00–1.00)	0.283
IL-1beta	1.00 (1.00–1.00)	0.234
IL-1RA	1.00 (1.00–1.00)	0.221
IL-4	1.00 (1.00–1.00)	0.284
IL-6	1.00 (1.00–1.00)	<0.001
IL-7	1.00 (1.00–1.00)	0.274
IL-8	1.00 (1.00–1.00)	0.111
IL-10	1.00 (1.00–1.00)	0.298
IL-17A	1.00 (1.00–1.00)	0.255
IP-10	1.00 (1.00–1.00)	0.589
MCP-1	1.00 (1.00–1.00)	0.024
MIP-1alpha	1.00 (1.00–1.00)	0.399
MIP-1beta	1.00 (0.99–1.00)	0.300
TNF-alpha	1.00 (1.00–1.00)	0.266
VEGF-A	1.00 (1.00–1.00)	0.363

Abbreviations: ACLF, acute-on-chronic liver failure; AD, acute decompensation; ALT, alanine aminotransferase; ; ASP, aspartate aminotransferase; BMI, body mass index; CI, confidence interval; G-CSF, granulocyte colony-stimulating factor; GIB, gastrointestinal bleeding; GM-CSF, granulocyte-macrophage colony-stimulating factor; HCV, hepatitis C virus; HR, hazard ratio; IFN, interferon; IL, interleukin; INR, international normalized ratio; IQR, interquartile range; MASLD, metabolic dysfunction-associated steatotic liver disease; MCP, monocyte chemoattractant protein; MIP, macrophage inflammatory protein; OHE, overt hepatic encephalopathy; TNF, tumor necrosis factor; SBP, spontaneous bacterial peritonitis; VEGF, vascular endothelial growth factor; WCC, white cell count.

**Table 4:** Multivariable Cox Regression Analysis of Factors Associated Mortality in the Total Cohort and in Patients With or Without OHE at Admission.

	Total cohort (n = 1231)	*P* value	No OHE (n = 1068)	*P* value	OHE (n = 205)	*P* value
	HR (95%CI)		HR (95%CI)		HR (95%CI)	
Female sex			0.57 (0.28–1.18)	0.132	6.42 (1.43–28.93)	0.015
Age	1.05 (1.03–1.08)	<0.001	1.04 (1.01–1.07)	0.005	1.17 (1.08–1.28)	<0.001
Etiology of cirrhosis, n (%)						
Alcohol			0.57 (0.27–1.20)	0.136		
HCV					0.07 (0.01–0.90)	0.041
MASLD						
Other						
Lactulose			1.85 (0.96–3.59)	0.067		
Rifaximin						
OHE	2.01 (1.14–3.53)	0.016	NA		NA	
ACLF	4.01 (2.31–6.95)	<0.001	4.72 (2.48–9.01)	<0.001	15.96 (3.38–75.25)	0.001
Electrolyte disorder					2.62 (0.88–7.81)	0.084
GIB						
Bacterial infection						
Alcohol use disorder			3.31 (1.45–7.54)	0.004		
WCC	1.12 (1.07–1.18)	<0.001	1.06 (1.00–1.13)	0.041	1.24 (1.12–1.36)	<0.001
Platelets	0.99 (0.99–1.00)	0.015				
Albumin					0.50 (0.22–1.14)	0.098
IL-6	1.00 (1.00–1.00)	<0.001	1.00 (1.00–1.00)	<0.001	1.00 (1.00–1.00)	0.003
IL-8	1.00 (1.00–1.01)	0.019			1.00 (1.00–1.01)	0.087
IL-10			1.00 (0.99–1.00)	0.415		
MCP-1						

Multivariable model selection was fitted by using a stepwise backward method based on Akaike Information criteria using all variables included in univariable analysis. Severity scores and original variables that are part of the ACLF definition were not included.

Abbreviations: ACLF, acute-on-chronic liver failure; CI, confidence interval; HR, hazard ratio; IL, interleukin; MASLD, metabolic dysfunction-associated steatotic liver disease; NA, not applicable; OHE, overt hepatic encephalopathy; WCC, white cell count.

**Figure 2:**
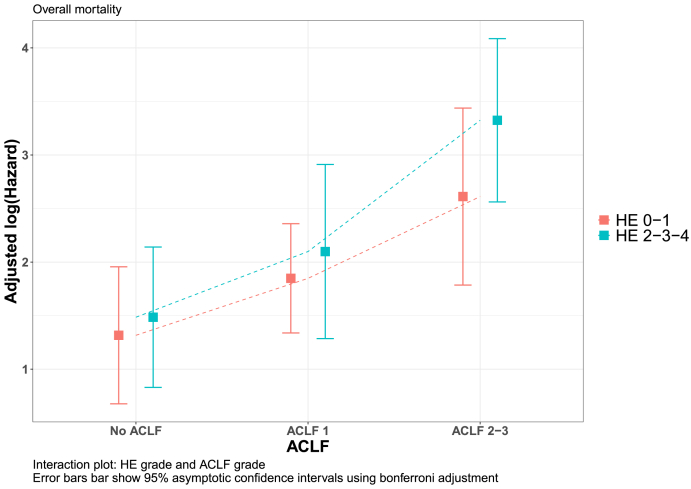
Interaction plot for overall survival between HE and ACLF grades adjusted by age. Results are given on the log (not the response) scale. Confidence level used: 0.95. *P* value adjustment: Tukey method for comparing a family of 6 estimates with significance level used: alpha = 0.05. ACLF, acute-on-chronic liver failure; HE, hepatic encephalopathy.

Considering that age and ACLF were associated with both OHE and risk of death and that OHE can be part of the ACLF definition, we performed a *post hoc* analysis of the interaction effect between ACLF and OHE adjusted by age. When we evaluated the risk of death according to the presence or absence of OHE across ACLF grades, no significant differences were observed between OHE and non-OHE patients in any of the ACLF categories, while mortality was higher with increasing ACLF grades (Supplementary Table 6, Figure 2).

## DISCUSSION

We describe for the first time the natural history of OHE in a large, prospective, multicentre study, which is one of the most frequent complications of cirrhosis, is associated with considerable morbidity and mortality and the treatment of which is an unmet clinical need.^1^, ^2^, ^3^, ^4^, ^5^ We determined factors of progression and resolution and demonstrated that presence of OHE and lack of response to therapy are associated with a high risk of death, but the main determinant of mortality of patients with OHE is the presence and severity of ACLF. These data are important to risk stratify patients for escalation of care, use of established treatments, and design of clinical trials of new therapies.

In this cohort of 1273 patients with cirrhosis from the PREDICT study, 16% were hospitalized with OHE. The proportion of patients hospitalized with HE in a previous European study from the CANONIC cohort was higher (34%), but it also included patients with HE grade 1 according to the West Haven criteria.^15^^,^^16^ This study included only patients with West Haven grade 2 or higher, which is currently defined as OHE because the diagnosis of HE grade 1 can be challenging in clinical practice leading to incorrect classification of patients.^15^

Previous history of HE was associated with the increased risk of hospitalization with OHE in our cohort and previous studies.^16^ Interestingly, patients receiving lactulose presented a higher risk of OHE in both univariable and multivariable analysis, probably as a surrogate of history of HE, but also showing that current secondary prophylactic approaches do not prevent patients from developing new HE episodes. This statement should be interpreted in light of the lack of compliance data in our cohort. In addition, previous treatment with lactulose was associated with poor response to treatment and higher risk of progression. Similarly, a study performed by Maggi DC *et al.* investigated factors associated with absence of improvement or new-onset HE during hospitalization for AD of cirrhosis, and showed that previous HE, Child–Pugh C and ACLF were independently associated with unfavorable progression.^6^ In that study, 31% of patients exhibited an unfavorable progression of HE, while in our cohort, only 5% of patients presented absence of improvement or progression of OHE. These differences may be explained by several reasons. First, in the previous study, patients with HE grade 1 were also included in the definition of unfavorable progression. Second, their outcomes were evaluated during the first 3 days of hospitalization while we assessed evolution of patients at week 1 increasing the time and likelihood of response to therapy. Finally, despite presenting similar disease severity scores (MELD of 17 and Child–Pugh of 9), a total of 25% of their patients presented with ACLF while in our study, ACLF was present in only 16% of the cohort.

Bacterial infection was the most frequent precipitating illness, it was more frequently seen in patients admitted with OHE and it was a risk factor for OHE progression, reinforcing the idea that infection must be actively searched and early managed in all patients admitted with OHE.^12^^,^^17^ This observation is in contradistinction to the study by Cordoba *et al.*, who did not find over representation of bacterial infection as a precipitating event for OHE.^16^ This difference may well be related to the inclusion of grade 1 HE in the diagnosis of OHE in that study. The presence of higher white blood cell counts and systemic inflammatory markers such as IL-6 in patients with OHE compared to those without OHE in our study and as independent risk factors for 28-day mortality supports the hypothesis that bacterial infection is an important precipitant of OHE. In addition, multiple concomitant precipitating factors were associated with a threefold increased risk of OHE at admission.

Surprisingly, factors traditionally defined as precipitating OHE such as GIB, electrolyte disorders or alcohol use disorders were not seen more frequently in OHE patients. This can be justified by the low incidence of GIB in our and previous cohorts,^16^ attributed to improvement in the prevention of this complication.^18^ Although electrolyte disorders are associated with OHE in previous studies,^19^ its presence was similar (32%) in patients with and without OHE in our cohort as it can also be associated with other complications of cirrhosis such as ascites or kidney failure and the use of diuretics.^20^^,^^21^

MASLD as the etiology of cirrhosis was associated with a higher risk of OHE. As previously described, patients with conditions associated with the metabolic syndrome present a higher risk of OHE development and lower response to therapy.^22^^,^^23^

Another important observation of our study is that age was an independent risk factor not only for hospitalization with OHE^24^^,^^25^ but also for OHE progression and overall mortality. In previous studies, the risk of developing HE in decompensated cirrhosis patients older than 55 years was 2.3 times higher than younger patients.^24^ In addition, older age has been associated with nonresponse to rifaximin in patients with minimal HE.^22^ Therefore, special attention and caution must be paid when managing patients with advanced age to minimize the risk of poor outcomes.

Regarding the risk of death, several studies have suggested that mortality is significantly higher in patients with OHE compared to those without OHE and it increases with HE grades, but whether this increased mortality from OHE is independent of the ACLF severity was not clarified.^6^^,^^14^^,^^26^, ^27^, ^28^ In the study by Cordoba *et al.*, risk of mortality of patients with ACLF was significantly higher than that of patients without ACLF, and in each subgroup, the mortality was higher in patients with HE.^14^ Similarly, in the study performed by Maggi *et al.*, the risk of death was higher in patients with unfavorable progression of HE in both groups of patients with or without ACLF.^6^ Nonetheless, the ACLF grade was not considered in any of these studies, and therefore, whether higher mortality of patients with HE was dependent on the presence of other non-HE organ failures is unclear. Overall mortality in our patients with OHE was similar to that reported previously (48% at 1 year) and it also increased with the HE grade; but the most important finding was that considering both OHE and ACLF grades and after adjusting by age, the effect of OHE on the risk of death seemed to be minimal within each ACLF grade while the most important factor that determined prognosis was the association with other organ failures. Moreover, ACLF was a risk factor for death in multivariable analysis in both patients with and without OHE at admission. Therefore, as shown in this study, it is essential to evaluate the presence of other organ failures in patients hospitalized with OHE, to adequately assess their risk of death and improve their prognosis.

The results of this study should be interpreted considering its strengths and limitations. First, the primary aim of the PREDICT study was not to evaluate OHE but to predict ACLF development. Therefore, patients with ACLF at admission were not followed up at 1 week and whether ACLF has an impact on the progression or resolution of OHE could not be evaluated. Second, only acute OHE leading to hospitalization was considered, not including non-acute decompensations of cirrhosis managed in the outpatients setting.^29^ Nonetheless, this is a prospective study including patients from 48 European centres, where findings are based on a well-characterized patient cohort, increasing the likelihood of applicability in the real world. Third, some factors that may influence OHE prognosis such as presence of transjugular intrahepatic portosystemic shunt or spontaneous portosystemic shunts, sarcopenia, or frailty were not considered in the study. Finally, the role of ammonia in the development and progression of OHE could not be evaluated as ammonia measurements were not protocolized as part of the study.^30^

In summary, the results of this study demonstrate that age, MASLD cirrhosis, previous treatment with lactulose, presence of ACLF, white cell count, and albumin levels at admission are associated with OHE. Age and treatment with lactulose, as well as presence of bacterial infections are risk factors for OHE progression and nonresponse to therapy. In hospitalized cirrhosis patients without ACLF, dynamic changes of OHE at week 1 have prognostic impact. Presence of other organ failures and non-recovery from OHE are associated with a high short-term mortality rate, while resolution of OHE is associated with significantly better prognosis. Understanding the natural history of patients with an acute episode of OHE and the factors defining prognosis and response to therapy will have profound implications on the development of novel approaches to the management of this debilitating illness.

## CRediT Authorship Contribution Statement

Statistical analyses were performed by JAC and MPB. The manuscript was prepared and written by MBP, JAC, AB, CR, and RJ. FA, FF, CS, RM, VA, SM, MST, VV, JG, VL, JC, and JT contributed to data collection. All authors have reviewed and approved the final submitted manuscript.

## Funding

MPB is supported by a Juan Rodés contract (JR23/00029), Instituto de Salud Carlos III. This work was supported in part by Instituto de Salud Carlos III (PI23/00062) to MPB. Funders were not involved in study design, data collection, analysis, decision to publish, or preparation of the manuscript.

## Declaration of Competing Interest

Rajiv Jalan is the founder of Yaqrit Discovery, a spin out company from University College London, Hepyx Limited and Cyberliver. He had research collaborations with Yaqrit Discovery. The other authors have no conflicts of interest to declare.
