# Supplementary material for: Factors Associated With Progression, Resolution and Mortality of Patients With Overt Hepatic Encephalopathy
Source: J Clin Exp Hepatol. 2025 Nov 7;16(1):103410. doi: 10.1016/j.jceh.2025.103410 (PMC12720073; doi:10.1016/j.jceh.2025.103410)
Supplement: Multimedia component 1 [file mmc1.pdf]

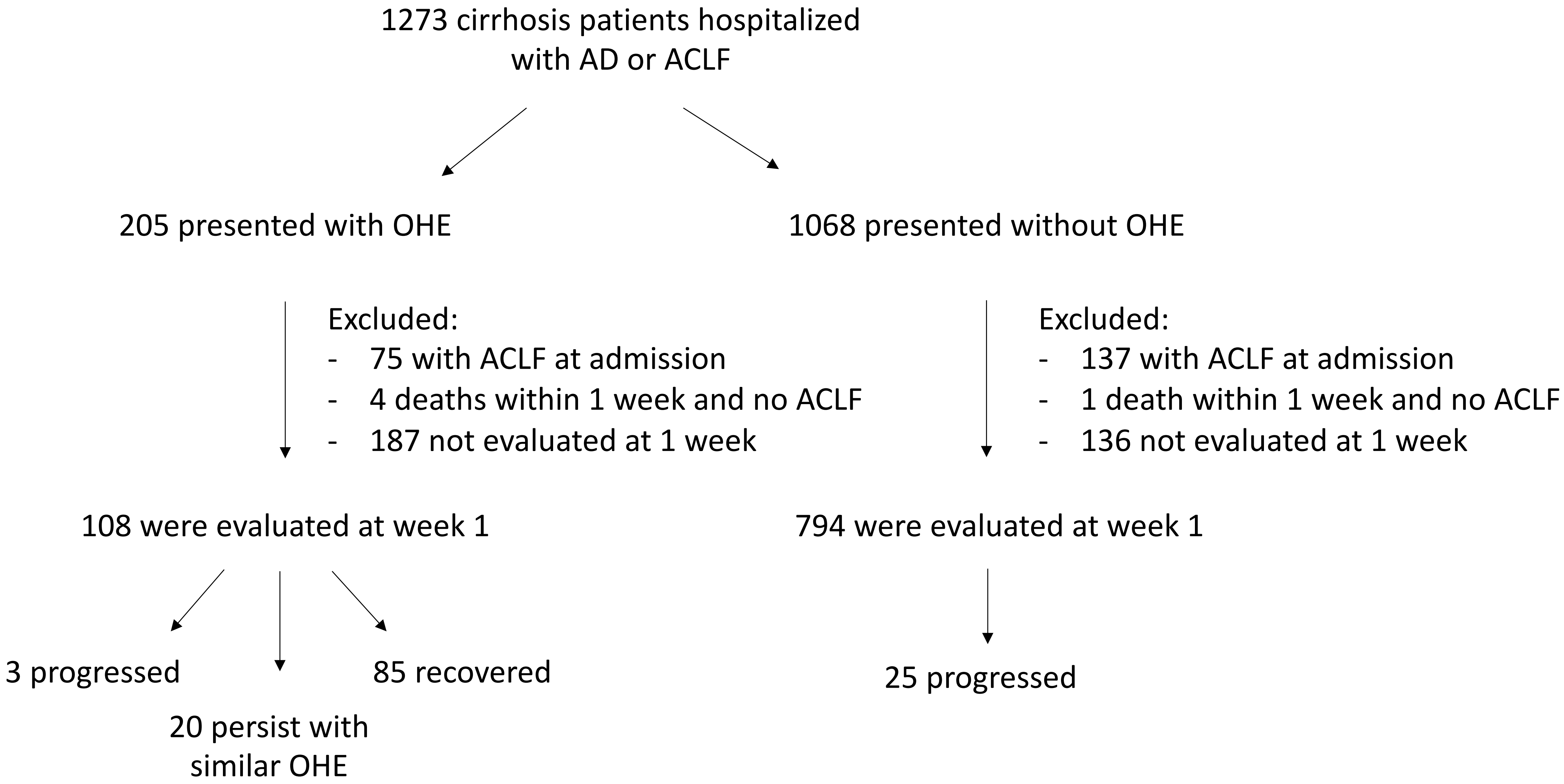
1273 cirrhosis patients hospitalized with AD or ACLF
205 presented with OHE
1068 presented without OHE
Excluded:
- 75 with ACLF at admission
- 4 deaths within 1 week and no ACLF
- 187 not evaluated at 1 week
Excluded:
- 137 with ACLF at admission
- 1 death within 1 week and no ACLF
- 136 not evaluated at 1 week
108 were evaluated at week 1
794 were evaluated at week 1
3 progressed
20 persist with similar OHE
85 recovered
25 progressed
