# Supplementary material for: Factors Associated With Progression, Resolution and Mortality of Patients With Overt Hepatic Encephalopathy
Source: J Clin Exp Hepatol. 2025 Nov 7;16(1):103410. doi: 10.1016/j.jceh.2025.103410 (PMC12720073; doi:10.1016/j.jceh.2025.103410)
Supplement: Multimedia component 2 [file mmc2.docx]

**Factors associated with progression, resolution and mortality of patients with overt hepatic encephalopathy**

**Supplementary material:**

- Definitions.
- Measurement of inflammatory markers.
- Tables.
- Figures.
- References.

**Definitions**

- Electrolyte disorder was considered when sodium was lower than 130mEq/L or higher than 145mEq/L or potassium was lower than 3.5mEq/L or higher than 5.5mEq/L (1). An additional analysis of hyponatremia (sodium was lower than 130mEq/L) or hypokalemia (potassium was lower than 3.5mEq/L) were performed separately.
- Diagnostic criteria for acute decompensation were based on the development of ascites, hepatic encephalopathy, gastrointestinal bleeding, infection, or any combination of these (1):
  - Ascites was considered as the development or acute progression to grade 2 to 3 ascites, according to the International Ascites Club Classification. Patients with recurrent or refractory ascites who presented to hospital frequently for therapeutic paracentesis due to rapid reaccumulation of large ascites were not included in this definition.
  - Overt hepatic encephalopathy defined as West Haven ≥2 (2) was considered as the acute development of a change in mental status in a patient with previous normal consciousness and no evidence of an acute neurologic disease.
  - Gastrointestinal bleeding was considered as bleeding from an oesophageal, gastric, or ectopic varix at the time of endoscopy, or the presence of large varices with blood in the lumen and no other recognisable cause of bleeding (3).
  - Infection was considered when there were clinical signs of infection [fever ≥38°C, leucocytosis (white blood cell count ≥12,000/mm) or requiring [antibiotic therapy](https://www.sciencedirect.com/topics/medicine-and-dentistry/antibiotic-therapy)] or positive confirmatory test such as polymorphonuclear count in ascitic/pleural fluid ≥250/mm^3^, infiltrates on chest x-ray or positive cultures (urinary, blood, sputum, cutaneous, stool) (4).
- ACLF definition was based on the European definition (5):
  - Both patients with prior decompensation and those without were included.
  - Failure of the liver, kidney, brain, coagulation, circulation and/or respiration were considered as defined by the CLIF-C OF scoring system.
  - Brain failure was defined as West Haven ≥3 (2).
- Liver disease severity was evaluated using the Child-Pugh (6), MELD (7), CLIF-AD (8) and CLIF-ACLF scores (9).
- Alcohol use disorder was defined according to the DSM-V criteria (10).

**Measurement of inflammatory markers**

The plasma levels of cytokines, chemokines and growth factors were determined by high-throughput multiplex assays using the Luminex® platform. Data analysis was performed using the Belysa immunoassay curve-fitting software. A five-parameter logistic regression model was used to generate standards curves (pg/mL) and to calculate the concentration of each sample.

**Tables**

**Supplementary Table 1.** Univariable and multivariable logistic regression analysis of factors associated with OHE at admission and with progression of OHE at week 1 replacing electrolyte disorder for hyponatremia and hypokalemia.

| **Parameter** | **Univariable** | | **Multivariable** | |
| --- | --- | --- | --- | --- |
|  | **OR (95%CI)** | **p-value** | **OR (95%CI)** | **p-value** |
| **Presence of OHE at admission (n=1273)** | | | | |
| Female sex | 0.63 (0.35-1.09) | 0.108 | 0.65 (0.34-1.17) | 0.161 |
| Age | 1.03 (1.00-1.05) | 0.021 | 1.03 (1.01-1.06) | 0.014 |
| Etiology of cirrhosis, n (%)   - Alcohol - HCV - MASLD - Other | 0.83 (0.50-1.41)  0.79 (0.27-1.90)  2.41 (1.11-4.87) | 0.484  0.636  0.018 | 2.67 (1.12-6.05) | 0.021 |
| Lactulose | 4.50 (2.51-8.58) | <0.001 | 3.97 (2.12-7.87) | <0.001 |
| Rifaximin | 2.32 (1.34-3.94) | 0.002 | 1.61 (0.87-2.92) | 0.123 |
| Hyponatremia | 1.42 (0.78-2.49) | 0.239 |  |  |
| Hypokalemia | 0.88 (0.47-1.58) | 0.689 |  |  |
| GIB | 0.72 (0.31-1.49) | 0.411 |  |  |
| Bacterial infection | 1.64 (1.00-2.71) | 0.051 |  |  |
| Alcohol use disorder | 0.69 (0.31-1.37) | 0.318 |  |  |
| ACLF at admission | 3.64 (2.05-6.33) | <0.001 | 2.44 (1.28-4.58) | 0.006 |
| WCC | 1.07 (1.03-1.12) | 0.001 | 1.08 (1.03-1.14) | 0.002 |
| Platelets | 1.00 (0.99-1.00) | 0.120 |  |  |
| Albumin | 0.60 (0.39-0.91) | 0.018 | 0.54 (0.33-0.85) | 0.010 |
| IL-6 | 1.00 (1.00-1.00) | 0.069 |  |  |
| IL-8 | 1.01 (1.00-1.01) | 0.010 |  |  |
| IL-10 | 1.00 (1.00-1.00) | 0.020 |  |  |
| MCP-1 | 1.00 (1.00-1.00) | 0.001 |  |  |
| **Progression of OHE at week 1 (n=902)** | | | | |
| Female sex | 1.55 (0.49-4.74) | 0.441 |  |  |
| Age | 1.05 (0.99-1.10) | 0.100 | 1.05 (0.99-1.12) | 0.106 |
| Etiology of cirrhosis, n (%)   - Alcohol - HCV - MASLD - Other | 0.91 (0.30-3.05)  0.89 (0.05-4.76)  0.84 (0.05-4.45) | 0.866  0.915  0.867 |  |  |
| Lactulose | 3.28 (0.99-14.80) | 0.074 | 3.96 (1.12-18.97) | 0.049 |
| Rifaximin | 0.72 (0.11-2.75) | 0.674 |  |  |
| Hyponatremia | 2.16 (0.57-6.85) | 0.211 |  |  |
| Hypokalemia | 0.59 (0.09-2.25) | 0.500 |  |  |
| GIB | 0.54 (0.03-2.81) | 0.554 |  |  |
| Bacterial infection | 2.42 (0.79-7.65) | 0.120 | 2.54 (0.76-8.64) | 0.124 |
| Alcohol use disorder | 1.67 (0.37-5.64) | 0.447 |  |  |
| WCC | 0.94 (0.79-1.08) | 0.417 |  |  |
| Platelets | 1.00 (0.99-1.00) | 0.296 |  |  |
| INR | 1.06 (0.26-1.85) | 0.899 |  |  |
| Bilirubin | 1.05 (0.94-1.14) | 0.285 |  |  |
| Albumin | 1.54 (0.62-3.59) | 0.334 |  |  |
| Creatinine | 0.82 (0.16-3.54) | 0.805 |  |  |
| IL-6 | 1.00 (1.00-1.00) | 0.099 | 1.00 (1.00-1.01) | 0.017 |
| IL-8 | 1.01 (0.99-1.02) | 0.330 | 1.02 (1.00-1.03) | 0.024 |
| IL-10 | 1.00 (0.98-1.00) | 0.867 |  |  |
| MCP-1 | 1.00 (0.99-1.00) | 0.690 | 0.99 (0.99-1.00) | 0.043 |

Severity scores and original variables that are part of the ACLF definition or electrolyte disorder were not included.

Abbreviations: metabolic dysfunction-associated steatotic liver disease (MASLD), acute-on-chronic liver failure (ACLF), gastrointestinal bleeding (GIB), overt hepatic encephalopathy (OHE), white cell count (WCC), international normalized ratio (INR), interleukin (IL), monocyte chemoattractant protein (MCP).

**Supplementary Table 2.** Univariable and multivariable logistic regression analysis of factors associated with OHE at admission and with progression of OHE at week 1 according to the presence of multiple precipitanting factors at admission.

| **Parameter** | **Univariable** | | **Multivariable** | |
| --- | --- | --- | --- | --- |
|  | **OR (95%CI)** | **p-value** | **OR (95%CI)** | **p-value** |
| **Presence of OHE at admission (n=1273)** | | | | |
| Female sex | 0.61 (0.34-1.06) | 0.088 | 0.62 (0.33-1.13) | 0.128 |
| Age | 1.03 (1.01-1.05) | 0.017 | 1.04 (1.02-1.07) | 0.003 |
| Etiology of cirrhosis, n (%)   - Alcohol - HCV - MASLD - Other | 0.88 (0.53-1.48)  0.75 (0.25-1.81)  2.35 (1.09-4.74) | 0.613  0.566  0.022 | 3.67 (1.48-8.82) | 0.004 |
| Lactulose | 4.67 (2.61-8.90) | <0.001 | 4.21 (2.22-8.44) | <0.001 |
| Rifaximin | 2.38 (1.39-34.01) | 0.001 | 1.83 (0.99-3.36) | 0.053 |
| Multiple precipitanting factors | 2.60 (1.58-4.30) | <0.001 | 3.08 (1.72-5.63) | <0.001 |
| ACLF at admission | 3.74 (2.13-6.46) | <0.001 | 2.11 (1.10-3.98) | 0.022 |
| WCC | 1.08 (1.03-1.12) | 0.001 | 1.09 (1.03-1.14) | 0.001 |
| Platelets | 1.00 (0.99-1.00) | 0.103 |  |  |
| Albumin | 0.62 (0.41-0.94) | 0.024 | 0.59 (0.36-0.94) | 0.030 |
| IL-6 | 1.00 (1.00-1.00) | 0.061 |  |  |
| IL-8 | 1.01 (1.00-1.01) | 0.012 |  |  |
| IL-10 | 1.00 (1.00-1.00) | 0.021 |  |  |
| MCP-1 | 1.00 (1.00-1.00) | 0.001 |  |  |
| **Progression of OHE at week 1 (n=902)** | | | | |
| Female sex | 1.57 (0.50-4.80) | 0.429 |  |  |
| Age | 1.04 (0.99-1.10) | 0.103 | 1.05 (0.99-1.11) | 0.135 |
| Etiology of cirrhosis, n (%)   - Alcohol - HCV - MASLD - Other | 0.91 (0.30-3.05)  0.87 (0.05-4.64)  0.85 (0.05-4.49) | 0.873  0.897  0.873 |  |  |
| Lactulose | 3.30 (0.99-14.88) | 0.073 | 3.71 (1.07-17.53) | 0.057 |
| Rifaximin | 0.73 (0.11-2.78) | 0.683 |  |  |
| Multiple precipitanting factors | 1.75 (0.48-6.43) | 0.385 |  |  |
| WCC | 0.94 (0.79-1.07) | 0.403 |  |  |
| Platelets | 1.00 (0.99-1.00) | 0.299 |  |  |
| INR | 1.06 (0.26-1.86) | 0.893 |  |  |
| Bilirubin | 1.05 (0.94-1.13) | 0.332 |  |  |
| Albumin | 1.54 (0.62-3.59) | 0.335 |  |  |
| Creatinine | 0.82 (0.15-3.53) | 0.797 |  |  |
| IL-6 | 1.00 (1.00-1.00) | 0.099 | 1.01 (1.00-1.01) | 0.010 |
| IL-8 | 1.01 (0.99-1.02) | 0.326 | 1.02 (1.00-1.03) | 0.031 |
| IL-10 | 1.00 (0.98-1.00) | 0.867 |  |  |
| MCP-1 | 1.00 (0.99-1.00) | 0.690 | 0.99 (0.99-1.00) | 0.045 |

Severity scores and original variables that are part of the ACLF definition or electrolyte disorder were not included.

Abbreviations: metabolic dysfunction-associated steatotic liver disease (MASLD), acute-on-chronic liver failure (ACLF), gastrointestinal bleeding (GIB), overt hepatic encephalopathy (OHE), white cell count (WCC), international normalized ratio (INR), interleukin (IL), monocyte chemoattractant protein (MCP).

**Supplementary Table 3.** Multivariable logistic-regression analysis of factors associated with OHE at admission in patients with and without prior HE.

|  | **Prior HE (n=93)** |  | **No prior HE (n=343)** |  |
| --- | --- | --- | --- | --- |
|  | **OR (95%CI)** | **p-value** | **OR (95%CI)** | **p-value** |
| Female sex |  |  | 0.26 (0.06-0.78) | 0.033 |
| Age |  |  | 1.07 (1.03-1.12) | 0.003 |
| Etiology of cirrhosis, n (%)   - Alcohol - HCV - MASLD - Other | 4.64 (0.90-26.87)  7.75 (1.71-42.85) | 0.068  0.010 |  |  |
| Lactulose |  |  |  |  |
| Rifaximin |  |  |  |  |
| ACLF |  |  |  |  |
| Hyponatremia |  |  |  |  |
| Hypokalemia |  |  |  |  |
| GIB | 3.19 (0.66-17.93) | 0.156 |  |  |
| Bacterial infection |  |  |  |  |
| Alcohol use disorder |  |  |  |  |
| WCC | 1.17 (1.07-1.31) | 0.002 |  |  |
| Platelets |  |  |  |  |
| Albumin |  |  | 0.45 (0.19-0.98) | 0.052 |
| IL-6 |  |  |  |  |
| IL-8 |  |  |  |  |
| IL-10 |  |  |  |  |
| MCP-1 |  |  |  |  |

Severity scores and original variables that are part of the ACLF definition were not included.

Abbreviations: metabolic dysfunction-associated steatotic liver disease (MASLD), acute-on-chronic liver failure (ACLF), overt hepatic encephalopathy (OHE), white cell count (WCC), interleukin (IL), not applicable (NA).

**Supplementary Table 4.** Characteristics of patients according to OHE progression at week 1.

| Parameter | No progression (n=874) | Progression (n=28) | p-value |
| --- | --- | --- | --- |
| Male sex, n (%) | 583 (67) | 18 (64) | 0.789 |
| Age, median (IQR) | 59 (52-67) | 64 (53-69) | 0.176 |
| Etiology of cirrhosis, n (%)   - Alcohol - HCV - Alcohol + HCV - MASLD - Other | 475 (64)  52 (6)  42 (5)  72 (9)  233 (27) | 16 (64)  3 (11)  1 (4)  1 (4)  7 (25) | 0.973  0.414  1.000  0.723 |
| Previous history (3-month), n (%)   - Ascites - HE - GIB - Hospitalization for infection - SBP - ACLF | 394 (57)  143 (22)  85 (13)  100 (16)  38 (6)  25 (4) | 12 (52)  8 (36)  4 (18)  7 (32)  1 (5)  2 (9) | 0.673  0.125  0.518  0.072  1.000  0.248 |
| Treatments, n (%)   - Lactulose - Rifaximin | 470 (54)  196 (22) | 21 (75)  8 (29) | 0.026  0.444 |
| Precipitating illness at admission, n (%)   - Electrolyte disorder - GIB - Bacterial infection - Alcohol use disorder | 248 (28)  123 (14)  251 (29)  119 (14) | 11 (39)  1 (4)  15 (54)  9 (32) | 0.414  0.160  0.005  0.011 |
| Child-Pugh class, n (%)   - A - B - C - Unknown | 61 (7)  412 (47)  323 (37)  78 (9) | 0 (0)  11 (39)  15 (54)  2 (7) | 0.221 |
| Child-Pugh, median (IQR) | 9 (8-10) | 9 (7-10) | 0.009 |
| MELD, median (IQR) | 15 (12-19) | 18 (14-20) | 0.029 |
| MELD-Na, median (IQR) | 19 (15-23) | 22 (16-25) | 0.041 |
| CLIF-AD score, median (IQR) | 52 (47-57) | 56 (50-61) | 0.012 |
| Biochemical parameters, median (IQR)   - WCC (x10^9/L) - Platelets (x10^9/L) - INR - Prothrombin time (seg) - Albumin (g/dL) - AST (U/L) - ALT (U/L) - Bilirubin (mg/dL) - Creatinine (mg/dL) - Sodium (mEq/L) - Potassium (mEq/L) - C reactive protein (mg/L) | 6.4 (4.3-9.1)  101 (64-151)  1.4 (1.3-1.7)  16 (14-21)  2.8 (2.5-3.3)  54 (34-86)  29 (18-45)  2.6 (1.4-5.5)  0.9 (0.7-1.2)  136 (132-138)  4.0 (3.6-4.4)  17 (7-37) | 7.3 (4.6-8.9)  92 (66-113)  1.6 (1.4-1.7)  17 (15-21)  2.7 (2.5-3.1)  62 (39-87)  29 (19-52)  3.8 (2.1-6.9)  0.8 (0.8-1.1)  135 (132-138)  4.1 (3.6-4.5)  32 (14-44) | 0.389  0.284  0.026  0.280  0.482  0.351  0.510  0.033  0.962  0.602  0.746  0.151 |
| Inflammatory markers (pg/mL), median (IQR)   - Eotaxin - G-CSF - GM-CSF - IFN-alpha2 - INF-gamma - IL-1alpha - IL-1beta - IL-1RA - IL-4 - IL-6 - IL-7 - IL-8 - IL-10 - IL-17A - IP-10 - MCP-1 - MIP-1alpha - MIP-1beta - TNF-alpha - VEGF-A | 80 (56-114)  15 (5-40)  10 (2-29)  15 (5-33)  26 (9-67)  2.7 (0.9-6.4)  4.6 (2.0-11.2)  3.6 (1.9-7.3)  1.6 (0.4-4.7)  9.8 (4.8-21.1)  1.1 (0.4-3.5)  3.8 (1.5-9.9)  6.1 (2.1-15.1)  2.7 (1.0-7.1)  190 (116-334)  164 (117-220)  11.8 (5.5-23.4)  15.3 (11.4-20.5)  22.3 (13.6-39.9)  5.0 (1.6-12.8) | 88 (65-98)  23 (15-87)  6 (4-11)  17 (5-22)  19 (9-87)  2.2 (0.5-6.9)  5.6 (1.5-10.9)  3.0 (1.6-5.7)  5.2 (0.4-50)  12.0 (6.4-43.4)  1.4 (0.4-17.7)  3.7 (1.4-9.4)  7.2 (3.7-31.9)  2.6 (0.5-6.9)  173 (101-336)  141 (90-202)  11.6 (4.8-16.8)  16.7 (10.6-24.9)  24.3 (13.9-47.7)  4.0 (1.2-12.3) | 0.983  0.122  0.479  0.781  0.837  0.864  0.970  0.461  0.343  0.075  0.571  0.900  0.265  0.920  0.761  0.106  0.856  0.490  0.723  0.788 |

Abbreviations: acute decompensation (AD), acute-on-chronic liver failure (ACLF), body mass index (BMI), gastrointestinal bleeding (GIB), hepatitis C virus (HCV), overt hepatic encephalopathy (OHE), metabolic dysfunction-associated steatotic liver disease (MASLD), spontaneous bacterial peritonitis (SBP), white cell count (WCC), international normalized ratio (INR), aspartate aminotransferase (ASP), alanine aminotransferase (ALT), granulocyte colony-stimulating factor (G-CSF), granulocyte-macrophage colony-stimulating factor (GM-CSF), interferon (IFN), interleukin (IL), monocyte chemoattractant protein (MCP), macrophage inflammatory protein (MIP), tumor necrosis factor (TNF), vascular endothelial growth factor (VEGF).

**Supplementary Table 5.** Characteristics of patients according to OHE resolution at week 1.

| Parameter | No resolution (n=23) | Resolution (n=85) | p-value |
| --- | --- | --- | --- |
| Male sex, n (%) | 16 (70) | 62 (73) | 0.748 |
| Age, median (IQR) | 61 (56-72) | 62 (56-71) | 0.955 |
| Race, n (%)   - Caucasian - Black - Asian - Other | 23 (100)  0 (0)  0 (0)  0 (0) | 82 (97)  0 (0)  1 (1)  2 (2) | 0.659 |
| Etiology of cirrhosis, n (%)   - Alcohol - HCV - Alcohol + HCV - MASLD - Other | 11 (58)  2 (10)  3 (13)  2 (10)  5 (21) | 43 (61)  4 (5)  3 (4)  12 (16)  22 (25) | 0.780  0.597  0.112  0.728 |
| Previous history (3-month), n (%)   - Ascites - HE - GIB - Hospitalization for infection - SBP - ACLF | 11 (58)  16 (76)  1 (5)  5 (25)  1 (5)  0 (0) | 42 (60)  39 (53)  5 (7)  10 (15)  6 (9)  2 (3) | 0.868  0.062  1.000  0.316  1.000  1.000 |
| Treatments, n (%)   - Lactulose - Rifaximin | 20 (87)  14 (61) | 73 (86)  38 (45) | 0.895  0.169 |
| HE grade at admission, n (%)   - 2 - 3 - 4 | 16 (70)  6 (26)  1 (4) | 72 (85)  12 (14)  1 (1) | 0.218 |
| Precipitating illness at admission, n (%)   - Electrolyte disorder - GIB - Bacterial infection - Alcohol use disorder | 6 (26)  2 (9)  7 (30)  3 (13) | 17 (20)  5 (6)  29 (34)  9 (11) | 0.527  0.639  0.740  0.716 |
| Child-Pugh class at admission, n (%)   - A - B - C - Unknown | 0 (0)  7 (30)  15 (65)  1 (4) | 2 (2)  40 (47)  41 (48)  2 (2) | 0.398 |
| Child-Pugh, median (IQR) | 11 (9-11) | 9 (8-11) | 0.060 |
| MELD, median (IQR) | 17 (15-20) | 15 (13-18) | 0.039 |
| MELD-Na, median (IQR) | 21 (17-25) | 18 (15-22) | 0.035 |
| CLIF-AD score, median (IQR) | 52 (50-59) | 54 (50-58) | 0.701 |
| Biochemical parameters, median (IQR)   - WCC (x10^9/L) - Platelets (x10^9/L) - INR - Prothrombin time (seg) - Albumin (g/dL) - AST (U/L) - ALT (U/L) - Bilirubin (mg/dL) - Creatinine (mg/dL) - Sodium (mEq/L) - Potassium (mEq/L) - C reactive protein (mg/L) | 5.2 (4.0-7.0)  79 (62-118)  1.6 (1.4-1.8)  18 (17-21)  2.7 (2.4-3.0)  60 (45-112)  40 (25-52)  3.8 (2.3-5.6)  0.8 (0.6-1.1)  135 (130-137)  4.1 (3.6-4.7)  10 (3-26) | 5.8 (4.4-8.4)  97 (60-133)  1.4 (1.3-1.7)  17 (14-21)  2.8 (2.6-3.1)  49 (33-72)  28 (18-44)  2.6 (1.6-4.8)  0.9 (0.7-1.3)  136 (132-139)  4.0 (3.7-4.4)  12 (6-37) | 0.246  0.297  0.034  0.200  0.305  0.092  0.044  0.079  0.163  0.302  0.982  0.430 |
| Inflammatory markers (pg/mL), median (IQR)   - Eotaxin - G-CSF - GM-CSF - IFN-alpha2 - INF-gamma - IL-1alpha - IL-1beta - IL-1RA - IL-4 - IL-6 - IL-7 - IL-8 - IL-10 - IL-17A - IP-10 - MCP-1 - MIP-1alpha - MIP-1beta - TNF-alpha - VEGF-A | 77 (57-113)  25 (15-42)  7 (1-76)  20 (11-44)  59 (8-97)  3.1 (1.5-11.5)  7.5 (2.3-15.0)  3.7 (1.9-8.0)  2.3 (0.9-11.9)  8.8 (5.1-22.7)  1.1 (0.5-3.5)  4.6 (1.7-7.5)  8.4 (3.6-22.7)  3.4 (1.0-13.5)  269 (113-370)  129 (103-184)  9.1 (6.0-28.4)  17.2 (13.4-28.4)  23.1 (11.8-36.5)  4.8 (0.4-28.0) | 86 (61-137)  16 (4-40)  5 (1-37)  14 (3-29)  26 (7-60)  2.1 (0.5-4.9)  4.6 (1.9-10.1)  3.3 (2.3-6.9)  1.6 (0.2-4.0)  8.0 (4.3-23.1)  0.5 (0.2-2.0)  3.6 (1.4-8.4)  7.5 (1.4-26.3)  2.3 (0.9-5.9)  228 (139-378)  164 (112-212)  11.2 (4.1-21.5)  15.2 (11.3-23.9)  25.7 (15.7-43.8)  6.1 (2.4-13.6) | 0.480  0.294  0.906  0.136  0.228  0.149  0.342  0.890  0.194  0.651  0.104  0.666  0.648  0.428  0.757  0.159  0.674  0.296  0.600  0.517 |

Abbreviations: acute decompensation (AD), acute-on-chronic liver failure (ACLF), body mass index (BMI), gastrointestinal bleeding (GIB), hepatitis C virus (HCV), overt hepatic encephalopathy (OHE), metabolic dysfunction-associated steatotic liver disease (MASLD), spontaneous bacterial peritonitis (SBP), white cell count (WCC), international normalized ratio (INR), aspartate aminotransferase (ASP), alanine aminotransferase (ALT), granulocyte colony-stimulating factor (G-CSF), granulocyte-macrophage colony-stimulating factor (GM-CSF), interferon (IFN), interleukin (IL), monocyte chemoattractant protein (MCP), macrophage inflammatory protein (MIP), tumor necrosis factor (TNF), vascular endothelial growth factor (VEGF).

**Supplementary Table 6.** Cox-regression interaction analysis of OHE and ACLF grades on the risk of death adjusted by age.

|  | ACLF grade | Log (Hazard) | Standard error | 95%CI | Group |
| --- | --- | --- | --- | --- | --- |
| Overall mortality | | | | | |
| No-OHE | No ACLF | 1.32 | 0.33 | 0.46-2.18 | a |
| OHE | No ACLF | 1.49 | 0.33 | 0.60-2.37 | ab |
| No-OHE | ACLF 1 | 1.85 | 0.26 | 1.16-2.53 | bc |
| OHE | ACLF 1 | 2.10 | 0.41 | 1.00-3.19 | abc |
| No-OHE | ACLF 2-3 | 2.62 | 0.42 | 1.50-3.72 | cd |
| OHE | ACLF 2-3 | 3.32 | 0.39 | 2.30-4.35 | d |

Conf-level adjustment: Bonferroni method for 6 estimates. Intervals are back-transformed from the logit scale. P value adjustment: Bonferroni method for 15 tests. Tests are performed on the log odds ratio scale. Significance level used: alpha = 0.05. If two or more means share the same grouping symbol, then we cannot show them to be different.

**Supplementary Table 7.** Inflammatory parameters according to the presence or absence of bacterial infection in OHE patients.

| Parameter (mean, SD) | No infection (n=123) | Infection (n=82) | p |
| --- | --- | --- | --- |
| Wcc (x10^9/L) | 7.6 (3.9) | 12.0 (7.8) | <0.001 |
| CRP (mg/L) | 22.0 (28.7) | 62.1 (55.3) | <0.001 |
| EOTAXIN (pg/ml) | 111.5 (82.5) | 116.5 (134.5) | 0.817 |
| G-CSF (pg/ml) | 408.1 (2273.0) | 216.3 (785.7) | 0.617 |
| GM-CSF (pg/ml) | 30.7 (35.7) | 375.8 (1292.0) | 0.197 |
| IFN-ALPHA2 (pg/ml) | 68.2 (230.0) | 89.0 (336.1) | 0.736 |
| IFN-GAMMA (pg/ml) | 66.2 (149.8) | 139.3 (502.7) | 0.320 |
| IL-1ALPHA (pg/ml) | 33.8 (182.6) | 158.1 (912.3) | 0.396 |
| IL-1BETA (pg/ml) | 28.6 (120.1) | 97.9 (569.0) | 0.393 |
| IL-1RA (pg/ml) | 14.2 (41.1) | 105.9 (453.6) | 0.138 |
| IL-4 (pg/ml) | 2.8 (3.7) | 1.8 (1.8) | 0.319 |
| IL-6 (pg/ml) | 134.6 (569.0) | 229.6 (596.9) | 0.411 |
| IL-7 (pg/ml) | 5.0 (14.8) | 11.6 (32.8) | 0.298 |
| IL-8 (pg/ml) | 27.7 (72.1) | 42.7 (145.0) | 0.499 |
| IL-10 (pg/ml) | 48.0 (158.9) | 142.7 (548.5) | 0.224 |
| IL-17A (pg/ml) | 22.5 (71.7) | 54.7 (285.7) | 0.520 |
| IP-10 (pg/ml) | 450.7 (972.6) | 616.7 (929.2) | 0.380 |
| MCP-1 (pg/ml) | 314.5 (527.0) | 346.1 (462.0) | 0.749 |
| MIP-1ALPHA (pg/ml) | 29.8 (57.1) | 25.5 (37.8) | 0.692 |
| MIP-1BETA (pg/ml) | 24.7 (18.4) | 24.9 (20.4) | 0.954 |
| TNF-ALPHA (pg/ml) | 49.7 (105.1) | 52.2 (69.8) | 0.889 |
| VEGF-A (pg/ml) | 17.9 (24.7) | 18.6 (32.1) | 0.910 |

**Figures**

- Supplementary Figure 1: Flowchart of the study population.

**References**

1. European Association for the Study of the Liver. EASL Clinical Practice Guidelines for the management of patients with decompensated cirrhosis. J Hepatol. 2018 Aug;69(2):406-460. doi: 10.1016/j.jhep.2018.03.024. Epub 2018 Apr 10. Erratum in: J Hepatol. 2018 Nov;69(5):1207. doi: 10.1016/j.jhep.2018.08.009. PMID: 29653741.
2. European Association for the Study of the Liver. Electronic address: easloffice@easloffice.eu; European Association for the Study of the Liver. EASL Clinical Practice Guidelines on the management of hepatic encephalopathy. J Hepatol. 2022 Sep;77(3):807-824. doi: 10.1016/j.jhep.2022.06.001. Epub 2022 Jun 17. Erratum in: J Hepatol. 2023 Nov;79(5):1340.
3. de Franchis R, Bosch J, Garcia-Tsao G, Reiberger T, Ripoll C; Baveno VII Faculty. Baveno VII - Renewing consensus in portal hypertension. J Hepatol. 2022 Apr;76(4):959-974. doi: 10.1016/j.jhep.2021.12.022. Erratum in: J Hepatol. 2022 Jul;77(1):271.
4. Piano S, Bunchorntavakul C, Marciano S, Rajender Reddy K. Infections in cirrhosis. Lancet Gastroenterol Hepatol. 2024 Aug;9(8):745-757. doi: 10.1016/S2468-1253(24)00078-5. Epub 2024 May 13. PMID: 38754453.
5. European Association for the Study of the Liver. EASL Clinical Practice Guidelines on acute-on-chronic liver failure. J Hepatol. 2023 Aug;79(2):461-491. doi: 10.1016/j.jhep.2023.04.021. Epub 2023 Jun 24. Erratum in: J Hepatol. 2024 Aug;81(2):370. doi: 10.1016/j.jhep.2024.03.012. PMID: 37364789.
6. Pugh RN, Murray-Lyon IM, Dawson JL, Pietroni MC, Williams R. Transection of the oesophagus for bleeding oesophageal varices. Br J Surg 1973; 60(8): 646-9.
7. Kamath PS, Wiesner RH, Malinchoc M, Kremers W, Therneau TM, Kosberg CL, et al. A model to predict survival in patients with end-stage liver disease. Hepatology. 2001 Feb;33(2):464-70. doi: 10.1053/jhep.2001.22172. PMID: 11172350.
8. Jalan R, Pavesi M, Saliba F, Amorós A, Fernandez J, Holland-Fischer P, et al; CANONIC Study Investigators; EASL-CLIF Consortium. The CLIF Consortium Acute Decompensation score (CLIF-C ADs) for prognosis of hospitalised cirrhotic patients without acute-on-chronic liver failure. J Hepatol. 2015 Apr;62(4):831-40. doi: 10.1016/j.jhep.2014.11.012. Epub 2014 Nov 22. Erratum in: J Hepatol. 2015 Jul;63(1):291. PMID: 25463539.
9. Jalan R, Saliba F, Pavesi M, Amoros A, Moreau R, Ginès P, et al; CANONIC study investigators of the EASL-CLIF Consortium. Development and validation of a prognostic score to predict mortality in patients with acute-on-chronic liver failure. J Hepatol. 2014 Nov;61(5):1038-47. doi: 10.1016/j.jhep.2014.06.012. Epub 2014 Jun 17. PMID: 24950482.
10. European Association for the Study of the Liver. EASL Clinical Practice Guidelines: Management of alcohol-related liver disease. J Hepatol. 2018 Jul;69(1):154-181. doi: 10.1016/j.jhep.2018.03.018. Epub 2018 Apr 5. PMID: 29628280.
